# *In Vitro* effects of petroleum ether, dichloromethane, methanolic and aqueous leaf extracts of *Eucalyptus grandis* on selected multidrug-resistant bacteria

**DOI:** 10.1371/journal.pone.0283706

**Published:** 2023-03-30

**Authors:** Bethel Kwansa-Bentum, Beatrice Awuradwoa Okine, Alberta D. Dayie, Patience B. Tetteh-Quarcoo, Fleischer C. N. Kotey, Eric S. Donkor, Nicholas T. K. D. Dayie

**Affiliations:** 1 Department of Animal Biology and Conservation Science, School of Biological Sciences, University of Ghana, Accra, Ghana; 2 Department of Chemistry, University of Cape Coast, Cape Coast, Ghana; 3 Department of Medical Microbiology, University of Ghana Medical School, Accra, Ghana; University of Jeddah, SAUDI ARABIA

## Abstract

**Background:**

The emergence and spread of antimicrobial resistance is of grave concern, requiring the search for newer and more effective antimicrobials to combat infections caused by resistant microbes. This study assessed the antimicrobial effects of *Eucalyptus grandis* crude extracts against selected multidrug resistant bacteria.

**Methodology:**

Four different crude leaf extracts of *E*. *grandis* were prepared using petroleum ether, dichloromethane, methanol, and water, with the aid of the Soxhlet extraction method. These were screened against methicillin-resistant *Staphylococcus aureus* (MRSA), multidrug resistant *Pseudomonas aeruginosa*, and multidrug resistant *Escherichia coli*, using the agar well diffusion method. Phytochemical screening was carried out to evaluate the bioactive phytochemical constituents responsible for the antimicrobial effect.

**Results:**

Each of the extracts, except for the one prepared from water, had antimicrobial activity against the screened bacteria. The non-polar petroleum ether extract had the highest antimicrobial activity (19.33–24.33 mm), including bactericidal effects, compared to the medium polar dichloromethane and polar methanol extracts, which recorded zone diameter ranges of 14.33–16.67 mm and 16.33–17.67 mm, respectively. The Gram-negative bacteria (*E*. *coli* and *P*. *aeruginosa*) were the least susceptible in comparison with the Gram-positive bacterium (MRSA), probably owing to differences in their cell wall structures. Furthermore, phytochemical screening indicated the presence of alkaloids, tannins, saponins, terpenoids, and flavonoids.

**Conclusion:**

The findings suggest that *E*. *grandis* could be potentially useful in the treatment of infections caused by multidrug resistant bacteria.

## Introduction

Treatment of infections is now hampered by a sustained high prevalence of multidrug resistance among circulating bacterial strains [1–14]. Infection with antibiotic resistant bacteria, such as multidrug resistant (MDR) *Mycobacterium tuberculosis*, vancomycin-resistant enterococci, and methicillin-resistant *Staphylococcus aureus* (MRSA), exert broad-ranged impacts that transcend health [15–19]. This has ignited a heightened interest in the exploration of sources of alternative antimicrobials, such as plants, that could be efficacious against multidrug-resistant pathogens [20–23].

The plant *Eucalyptus grandis* fits such a profile. It is native to Australia, where it is utilized in the treatment of sore throats and bacterial infections of the urinary, gastrointestinal, and respiratory tracts [24]. It is also widely available in Ghana and can be found at the frontage of the Centre for National Culture, Kumasi, whose indigenes believe it to be useful for the treatment of heart-related diseases, ulcer, boils, wounds, diseases of the urogenital system, liver, and kidney stones, among others. Although the plant has been revealed to contain phytochemicals such as tannins, glycosides, flavonoids, esters, and terpenes, and the bark of its stem is known to exhibit anti-*Helicobacter pylori* activity [25], the antimicrobial activity of its leaves has not been adequately explored. This study, therefore, investigated the effects of different crude extracts of *E*. *grandis* leaves on selected multidrug resistant bacteria to help expand the spectrum of antimicrobial agents from natural resources.

## Materials and methods

### Study design

The study was carried out between January and July 2015, and its design was experimental and explorative. Aqueous, methanol, dichloromethane and petroleum ether extracts of *E*. *grandis* were prepared at the Department of Chemistry, University of Ghana. Standard quality assurance processes were followed in the conduction of all the experiments. Assays for antimicrobial activity testing and determination of phytochemical components of the extracts were also performed using standard protocols. For each experiment, conventional antibiotics were used as control. Each experiment had three replicates, to eliminate errors and increase reliability of the outcome.

### Collection and preparation of plant material for extraction

Fresh samples of *E*. *grandis* leaves were collected and identified at the Ghana Herbarium, University of Ghana, where voucher specimens are deposited. The leaves were washed and rinsed under running distilled water. The water was allowed to drain from the leaves, after which the leaves were dried under a shade for two weeks. The dried samples were crushed mechanically using mortar and pestle, and finally reduced to fine particles using Waring Laboratory blender for five minutes under high speed.

### Preparation of crude extracts

Four different crude leaf extracts of *E*. *grandis* were prepared using petroleum ether, dichloromethane, methanol and water. The extracts obtained were concentrated using a Rotary evaporator that made the solvents boil at 40–50°C and dried in a desiccator. The residue was weighed and preserved until it was ready for use. The aqueous extraction is known to be one of the naturopathic treatment modalities used widely across the world [26].

### Soxhlet extraction

The method described by Wang and Weller [27] was used for the conventional Soxhlet system, with a slight modification. One hundred grams of the air-dried coarsely powered *E*. *grandis* leaves was placed in a thimble holder and filled with 150 mL of the condensed fresh solvent from its distillation flask. When the liquid reached its overflow level, the siphon aspirated the solution in the thimble holder and unloaded it back into the distillation flask, carrying the extracted solutes into the bulk liquid. In the solvent flask, the solute was separated from the solvent using distillation. The solute was left in the flask and the fresh solvent passed back into the plant solid bed. The operation was repeated until complete extraction was achieved.

### Preparation of aqueous extract

The aqueous extraction was carried out following the method described by Parekh & Chanda [28] with slight modifications. Twenty grams of air-dried powder was added to 150 mL of distilled water and boiled on slow heat for 2 hours. The resultant mixture was filtered through 8 layers of muslin cloth and centrifuged at 5000 g for 10 min, and the supernatant was collected. This procedure was repeated twice; after 6 hours, the supernatant was collected at an interval of 2 hours, pooled together and concentrated to make the final volume one-fourth of the original volume.

### Preparation of the diluted extracts

Various dilutions were obtained from the *E*. *grandis* extracts prepared by reconstituting each extract in 5% Dimethyl Sulfoxide (DMSO). This was then tested against the selected microorganisms, and pure 5% DMSO was used as a control. The concentration of the extracts used was 0.1 g/mL, and this was determined by dissolving 1g of each crude extract in 10 mL of 5% DMSO.

### Test organisms

The extracts were tested against MDR isolates, including one Gram positive bacterium, MRSA (NCTC 25923), and two Gram negative bacteria, *Escherichia coli* (NCTC 13351) and *Pseudomonas aeruginosa* (ATCC 27853), obtained from the stock cultures of the Department of Medical Microbiology, University of Ghana Medical School. These isolates were maintained on nutrient agar slopes, and their identities were confirmed following standard procedures; they were confirmed as MDR based on their resistance to at least, three classes of antibiotics. They were grown on nutrient agar plates and incubated at 37°C for 24 hours before testing.

### Antimicrobial activity testing of the crude extracts

Screening for antimicrobial activity of the crude *E*. *grandis* extracts was performed in triplicates, using the agar-well diffusion method. The extracts were allowed to diffuse out into the medium and interact in the plate freshly seeded with the test organisms; the inoculums had turbidities similar to that of 0.5 McFarland standard. With the aid of a sterile cork borer, wells (6 mm in diameter) were created in the seeded plates. The extracts were pipetted into each well, after which the plates were incubated at 37°C for 24 hours. The antimicrobial spectrum of the various extracts was determined in terms of the diameter recorded for each inhibition zone. Zones of inhibition were measured using a rule.

### Control set-up

A control experiment was set up by testing conventional antibiotics against the test organisms (MDR *Pseudomonas aeruginosa*, methicillin-resistant *Staphylococcus aureus*, and MDR *Escherichia coli*) using the disc plate method. The antibiotics included ampicillin, tetracycline, gentamicin, amoxicillin-clavulanic acid, sulphamethoxazole-trimethoprim, penicillin, ciprofloxacin, and cloxacillin (Oxoid Ltd., Basingstoke, UK). The antibiotic discs were placed on the surface of Mueller-Hinton agar plates that had been inoculated with the test microorganisms. During incubation, the antibiotics diffused outward from the discs thereby creating a concentration gradient. After 24 hours, each zone of inhibition was measured and recorded.

### Phytochemical testing

The phytochemical screening of all the extracts was carried out to determine the presence of the alkaloids, flavonoids, saponins, terpenoids and tannins, as described by Sofowora [29] with some modifications. For alkaloids, 1 mL of the extract was dissolved in distilled water, after which, 2 mL of Wagner’s reagent was added. The formation of a reddish-brown precipitate indicated the presence of alkaloids. For flavonoids, 1 mL of dilute ammonia solution was added to a 4 mL portion of the extract, followed by a few drops of concentrated H_2_SO_4_. A yellow colouration indicated the presence of flavonoids. For tannins, 1 g of each extract was dissolved in 20 mL of distilled water and boiled for 10 minutes. Three drops of 0.1% FeCl_3_ were added to the supernatant. A blue-black or brownish-green colouration indicated the presence of tannins. For saponins, 1 g of the extract was dissolved in 10 mL of distilled water and shaken vigorously for 15 minutes. The formation of a stable persistent froth indicated the presence of Saponins. For terpenoids, 1 mL of extract was dissolved in distilled water, followed by the addition of 1 mL chloroform and 1 mL of concentrated H_2_SO_4_. The formation of a reddish-brown colouration at the interface indicated the presence of terpenoids.

### Statistical analyses

Statistical analyses were done using Statistical Products and Services Solutions (SPSS), version 20. The zones of inhibition measured upon completion of the agar well diffusion assay were presented as means and standard deviations. The zones of inhibition recorded for the aqueous, petroleum ether, dichloromethane, and methanol extracts of *E*. *grandis* leaves, as well as for the DMSO, were compared using One-Way ANOVA, followed by Tukey HSD’s post-hoc test, at a 0.05 alpha level.

## Results

### Evaluation of the *Eucalyptus grandis* extract bioactivity

Except for the aqueous *E*. *grandis* extract, the *E*. *grandis* extracts obtained from all the solvents showed antimicrobial activity, and the zones of inhibition of these extracts ranged between 14.33 mm and 24.33 mm (Table 1). The differences in zones of inhibition across the five groups–aqueous (polar), petroleum ether (non-polar), dichloromethane (medium-polar), methanol (polar), distilled water (polar), and DMSO (the control)–were significant (*p* < 0.0001) (Table 2). The multiple comparisons of the zones of inhibition revealed the petroleum ether extract to have the strongest antimicrobial activity (*p* < 0.0001) (Table 3).

**Table 1:** Zones of inhibition of leaf extracts of *Eucalyptus grandis* on selected microorganisms.

Bacteria	Zone of inhibition of extracts (in millimeters [mm])				
	Aqueous (Polar)	Petroleum ether (Non-polar)	Dichloromethane (Medium-polar)	Methanol (Polar)	Dimethyl sulphate (Control)
**MRSA**	0	24.33 ± 1.53	16.67 ± 1.16	17.67 ± 1.53	0
***Escherichia coli***	0	20.33 ± 0.58	15.67 ± 1.53	17.33 ± 0.58	0
***Pseudomonas aeruginosa***	0	19.33 ± 1.53	14.33 ± 0.58	16.33 ± 0.58	0
**Combined**	0	21.33 ± 2.56	15.56 ± 1.42	17.11 ± 1.05	0

MRSA = Methicillin-resistant *Staphylococcus aureus*

**Table 2:** Analysis of variance of the zones of inhibition of the various *Eucalyptus grandis* extracts and the control.

	Sum of Squares	Df	Mean Square	F	*p* value
**Between Groups**	3660.089	4	915.022	474.651	< 0.0001
**Within Groups**	77.111	40	1.928		
**Total**	3737.200	44			

Df = Degree of freedom

**Table 3:** Multiple comparisons of the zones of inhibition recorded for the various *Eucalyptus grandis* extracts and the control.

(I) Extracts	(J) Extracts	Mean Difference (I-J)	Std. Error	*p* value	95% Confidence Interval	
					Lower Bound	Upper Bound
Aqueous	Petroleum ether	-21.333*	0.655	< 0.0001	-23.20	-19.46
	Dichloromethane	-15.556*	0.655	< 0.0001	-17.42	-13.69
	Methanol	-17.111*	0.655	< 0.0001	-18.98	-15.24
	DMSO	0.000	0.655	1.000	-1.87	1.87
Petroleum ether	Aqueous	21.333*	0.655	< 0.0001	19.46	23.20
	Dichloromethane	5.778*	0.655	< 0.0001	3.91	7.65
	Methanol	4.222*	0.655	< 0.0001	2.35	6.09
	DMSO	21.333*	0.655	< 0.0001	19.46	23.20
Dichloromethane	Aqueous	15.556*	0.655	< 0.0001	13.69	17.42
	Petroleum ether	-5.778*	0.655	< 0.0001	-7.65	-3.91
	Methanol	-1.556	0.655	0.143	-3.42	0.31
	DMSO	15.556*	0.655	< 0.0001	13.69	17.42
Methanol	Aqueous	17.111*	0.655	< 0.0001	15.24	18.98
	Petroleum ether	-4.222*	0.655	< 0.0001	-6.09	-2.35
	Dichloromethane	1.556	0.655	0.143	-0.31	3.42
	DMSO	17.111*	0.655	< 0.0001	15.24	18.98
DMSO (Control)	Aqueous	0.000	0.655	1.000	-1.87	1.87
	Petroleum ether	-21.333*	0.655	< 0.0001	-23.20	-19.46
	Dichloromethane	-15.556*	0.655	< 0.0001	-17.42	-13.69
	Methanol	-17.111*	0.655	< 0.0001	-18.98	-15.24

DMSO = Dimethyl sulphate; Std. Error = Standard Error

* The mean difference is significant at the 0.05 level

### Phytochemical analyses

The extracts that showed antimicrobial effects were subjected to phytochemical screening (Table 4). The investigations revealed the presence of alkaloids as a major phytochemical component in all the extracts, with tannins and terpenoids present only in the petroleum ether and dichloromethane extracts whilst saponins were only present in the methanol extract.

**Table 4:** Phytochemical screening of the different *Eucalyptus grandis* extracts that showed antimicrobial activity on the selected microorganisms.

Constituents	Extracts		
	Petroleum ether	Dichloromethane	Methanol
Alkaloids	+	+	+
Flavonoids	+	-	-
Tannins	+	+	-
Saponins	-	-	+
Terpenoids	+	+	-

Key: + = present;— = absent

## Discussion

In the present study, different crude extracts obtained from *E*. *grandis* leaves were evaluated to determine their bactericidal effect against multidrug resistant bacteria. Preliminary investigations showed that three of the different extracts of *E*. *grandis*–petroleum ether, dichloromethane, and methanol–were active against the selected microorganisms (methicillin-resistant *Staphylococcus aureus*, *Escherichia coli* and *Pseudomonas aeruginosa*). This analysis involving the use of several extracts to determine the efficacy of a plant material for antimicrobial activity has also been realized by many scientists in their search using plants like *Moringa oleifera*, *Elaeophorbia drupifera*, *Rauwolfia vomitoria*, *Eucalyptus globulus*, *Alchornea cordifolia* and *Lippia multiflora* [20, 30]. Among the different extracts used, petroleum ether showed the most significant antimicrobial activity against the multidrug resistant microorganisms. Although the mechanism of action of the plant constituents are not understood in its entirety, it is known that the efficacy of extracts, to a large extent, is contingent on the type of solvent used. The non-polar extracts had higher antimicrobial activity (19.33–24.33 mm) than did the medium polar (14.33–16.67 mm) and polar extracts (16.33–17.67 mm). The observations made indicate that the existence of non-polar residues in the extracts yielded higher bactericidal effects, compared to the medium polar and polar residues. Comparing the polar and medium polar extracts, methanol was seen to follow petroleum ether with regard to its high level of antimicrobial activity. This may be due to methanol being an organic solvent and its higher ability to dissolve organic matter organic nature of methanol and its high capacity to dissolve more organic and active antimicrobial compounds than the medium polar solvent, dichloromethane. The differences in polarities of the solvents may underly the disparities in the solubilities of the plant’s active principles [31]. The aqueous extract, on the other hand, known to be a polar solvent, showed no bactericidal activity, although water is known to be one of the naturopathic treatment modalities used worldwide. Perhaps the non-inhibitory effect is because the active ingredient in the leaves of *E*. *grandis* is hydrophobic.

The antimicrobial activity of the *E*. *grandis* extract showed more inhibitory effect on the Gram-positive MRSA, as compared to the Gram-negative bacteria. The lower complexity of the Gram-positive bacterial cell wall and its deficiency in sieving effect, in contrast with that of those Gram-negatives which have a high lipopolysaccharide content (and hence lower permeability), may account for this [32]. The results obtained in this study as far as susceptibility of Gram negative and Gram-positive bacteria are concerned complement other findings. The most resistant organism was found to be *P*. *aeruginosa*, as it showed the least zone of inhibition to any of the extracts used in this study. This could be due to the capsule, a polysaccharide layer outside the cell wall which is present in Gram negative bacteria, making it difficult for the extracts to penetrate.

The present study revealed that *E*. *grandis* leaves contain phytochemical constituents like terpenoids, tannins, saponins, flavonoids and alkaloids in the different solvent extracts, with petroleum ether extract having the most phytochemicals, thereby explaining why it had the highest antimicrobial activity. Alkaloids were present in all the extracts tested while flavonoids and saponins were observed only in the petroleum ether and methanol extracts, respectively. Notably, the metabolites/phytochemicals specify the biological effects [33, 34]. Flavonoids, for instance, are antiviral, antitoxic, and antitumour, among other things [35]. Also, terpenoids are widely relied on for their aromatic qualities, and they are known for their antibacterial and antineoplastic effects [36, 37]. Tannins also have bioactivity against parasites, bacteria, and viruses; saponins are known to cause haemolysis, and thus, making saponins good candidates for treatment of bacterial infections [38, 39]. Whereas these findings suggest that *E*. *grandis* can be considered in the search for new antimicrobial drugs, further studies need to be carried out to determine the minimum inhibitory concentration, erythrocytic toxicity levels as well as *in vivo* testing of the leaf extract.

## Conclusion

Various extracts of *E*. *grandis* (petroleum ether, dichloromethane, and methanol extracts) demonstrated significant inhibitory effects against multidrug resistant bacteria (MRSA, *Escherichia coli*, and *Pseudomonas aeruginosa*). The petroleum ether extract exhibited the highest antimicrobial activity, and thus holds a high potential for effective treatment of bacterial infections, as do the dichloromethane and methanol extracts. The widely used naturopathic treatment modality, the aqueous extract, showed no antibacterial activity, which may be because active components of the leaf are hydrophobic.

Future studies could determine the minimum inhibitory concentration (MIC) of the three promising extracts, as well as the erythrocytic toxicity levels. Moreover, *in vivo* testing of the leaf extracts needs to be carried out. Additionally, these subsequent studies could include performance of chromatography to identify the fractions of the crude extract that contain the compounds responsible for the observed antimicrobial activity. These compounds could then be isolated, and their structures elucidated.

## Supporting information

S1 Table Raw data on the zones of inhibition recorded following the agar well diffusion assay. (DOCX)
