# Supplementary material for: In Vitro effects of petroleum ether, dichloromethane, methanolic and aqueous leaf extracts of Eucalyptus grandis on selected multidrug-resistant bacteria
Source: PLoS One. 2023 Mar 30;18(3):e0283706. doi: 10.1371/journal.pone.0283706 (PMC10062571; doi:10.1371/journal.pone.0283706)
Supplement: S1 Table — (DOCX) [file pone.0283706.s001.docx]

**S1 Table. Raw data on the zones of inhibition (in millimeters) recorded following the agar well diffusion assay.**

| **Extracts/Bacteria** | **MRSA** | ***E. coli*** | ***P. aeruginosa*** |
| --- | --- | --- | --- |
| **Petroleum ether** | 24 | 20 | 18 |
|  | 26 | 20 | 21 |
|  | 23 | 21 | 19 |
| **Dichloromethane** | 16 | 14 | 15 |
|  | 16 | 16 | 14 |
|  | 18 | 17 | 14 |
| **Methanol** | 16 | 17 | 16 |
|  | 19 | 17 | 17 |
|  | 18 | 18 | 16 |
| **Aqueous** | 0 | 0 | 0 |
|  | 0 | 0 | 0 |
|  | 0 | 0 | 0 |
| **DMSO** | 0 | 0 | 0 |
|  | 0 | 0 | 0 |
|  | 0 | 0 | 0 |
